# RNA sequencing reveals differential long noncoding RNA expression profiles in bacterial and viral meningitis in children

**DOI:** 10.1186/s12920-024-01820-y

**Published:** 2024-02-12

**Authors:** Xin Li, Suzhen Sun, Huifeng Zhang

**Affiliations:** 1 Department of Pediatrics, The Second Hospital of Hebei Medical University, Hebei Medical University, No. 215 West Heping Street, Shijiazhuang, Hebei 050000 China; 2 First Department of Neurology, Hebei Children’s Hospital, Hebei Children’s Hospital Affiliated to Hebei Medical University, Shijiazhuang, 050000 China

**Keywords:** Bacterial meningitis, Viral meningitis, lncRNA, ceRNA

## Abstract

**Background:**

We aimed to investigate the involvement of long non-coding RNA (lncRNA) in bacterial and viral meningitis in children.

**Methods:**

The peripheral blood of five bacterial meningitis patients, five viral meningitis samples, and five healthy individuals were collected for RNA sequencing. Then, the differentially expressed lncRNA and mRNA were detected in bacterial meningitis vs. controls, viral meningitis vs. healthy samples, and bacterial vs. viral meningitis patients. Besides, co-expression and the competing endogenous RNA (ceRNA) networks were constructed. Receiver operating characteristic curve (ROC) analysis was performed.

**Results:**

Compared with the control group, 2 lncRNAs and 32 mRNAs were identified in bacterial meningitis patients, and 115 lncRNAs and 54 mRNAs were detected in viral meningitis. Compared with bacterial meningitis, 165 lncRNAs and 765 mRNAs were identified in viral meningitis. 2 lncRNAs and 31 mRNAs were specific to bacterial meningitis, and 115 lncRNAs and 53 mRNAs were specific to viral meningitis. The function enrichment results indicated that these mRNAs were involved in innate immune response, inflammatory response, and immune system process. A total of 8 and 1401 co-expression relationships were respectively found in bacterial and viral meningitis groups. The ceRNA networks contained 1 lncRNA-mRNA pair and 4 miRNA-mRNA pairs in viral meningitis group. GPR68 and KIF5C, identified in bacterial meningitis co-expression analysis, had an area under the curve (AUC) of 1.00, while the AUC of OR52K2 and CCR5 is 0.883 and 0.698, respectively.

**Conclusions:**

Our research is the first to profile the lncRNAs in bacterial and viral meningitis in children and may provide new insight into understanding meningitis regulatory mechanisms.

**Supplementary Information:**

The online version contains supplementary material available at 10.1186/s12920-024-01820-y.

## Introduction

Bacterial and viral meningitis are common inflammatory meningitis caused by bacteria and viruses [1]. Bacterial meningitis was an umbrella name caused by a diverse pathogenic bacterium. In neonates and children, *Escherichia coli*, *Listeria monocytogenes*, *Haemophilus infl uenzae type b*, *S pneumoniae*, and *Neisseria meningitidis* were responsible for bacterial meningitis [2]. Bacterial meningitis is an acute suppurative infection [3, 4]. Patients with bacterial meningitis are a severe medical emergency with a mortality of approximately 100% if left untreated. Despite optimal treatment, mortality and morbidity might happen [5]. On the contrary, the clinical manifestations of viral meningitis are mostly benign [3]. Even though, it is worth noting that severe complications can appear in neonates and children. *Enteroviruses* account for 23–61% of cases of viral meningitis [6]. Although there are many causes of meningitis, the manifestations of meningitis are very similar, like fever, headache, neck stiffness, nausea, and raised intracranial pressure [1, 7]. In addition, Bodkin et al. reported that the performance of published host gene expression signature in distinguishing between bacterial and viral infections does not differ dramatically [8]. Particularly, its performance was more poorly in pediatric samples [8]. Due to dangers of bacterial infection, differentiation between bacterial and viral meningitis, along with the urgent administration of targeted antimicrobial therapy, becomes imperative [3]. The potential pathogenesis of meningitis is the inflammatory reaction to the invading pathogen, which is responsible for the clinical symptoms [3, 4]. However, the accurate potential mechanisms of meningitis are still unclear, and the differentiation between bacterial and viral meningitis is not understood.

Long non-coding RNAs (lncRNAs), length more than 200 nt, attribute to a wide range of functions, containing modification of DNA, RNA, and histones, transcription, mRNA turnover, and translation [9, 10]. Previous researches have shown that lncRNAs play a role in specific pathophysiological phenotypes in response to bacterial meningitis in cell lines and animal models [11, 12]. Inhibition of lncRNA nuclear paraspeckle assembly transcript 1 (NEAT1) expression can increase miR-135a expression and reduce the blood-brain barrier (BBB) permeability in vitro bacterial meningitis-induced BBB damage models [12]. The lncRNA Morrbid activated CD 8 T cell to respond to interferon by enhancing the PI3K-AKT signaling pathway and modulating the proapoptotic factor (Bcl2l11) expression in viral infection [13]. Nevertheless, little is known about the action of lncRNAs during bacterial and viral meningitis in humans.

In this study, we applied high-throughput transcriptomics to explore the differentially expressed profile of lncRNAs and mRNAs among bacterial meningitis samples, viral meningitis patients, and healthy subjects and attempt to identify some potential molecular signatures to distinguish different meningitis. The function enrichment analysis and the possible correlation between lncRNAs and mRNAs were analyzed. Moreover, co-expression network and competing endogenous RNA (ceRNA) network were also constructed. In brief, our research is the first to profile the lncRNA and mRNA transcription involved in bacterial and viral meningitis in children, which may shed novel light on the regulator mechanisms of meningitis pathogenesis.

## Methods

### Subjects

A total of 8 viral meningitis and 5 bacterial meningitis subjects were recruited. The meningitis patients with the presence of bacterial antigen, bacteria, and viral nucleic acid in serum and cerebrospinal fluid were enrolled. Three cases of viral meningitis were not included in the cohort because their culture results were unclear. Samples with tuberculous meningitis, brain tumor, parenteral viral meningitis, and concurrent infection with meningitis and bacteremia were not enrolled [14]. Finally, 5 viral, 5 bacterial meningitis patients, and 5 healthy controls were enrolled in this study. All subjects ranged from one month to 16 years old, and all patients had clinical features of meningitis. A 5 ml volume of peripheral blood was collected and stored at -80 ℃. The study obtained informed consent from the parents of patients and passed an ethical review. The five bacterial meningitis subjects were labelled B 1, B 2, B 3, B 4, and B 5. Viral meningitis and control groups were marked similarly. Detailed information about all samples was displayed in Tables 1 and 2.

**Table 1:** Clinical presentation of 15 cases

Case no.	Age (years)	Gender	Fever	Headache	Spasms	Somnolence	Vomiting
B 1	3	Male	+	-	+	+	+
B 2	4	Female	+	-	-	+	-
B 3	10	Male	+	+	-	-	+
B 4	< 1	Female	+	-	+	+	+
B 5	< 1	Male	+	-	+	-	+
V 1	5	Female	+	+	-	+	-
V 2	5	Male	+	-	-	-	+
V 3	4	Male	+	+	-	-	-
V 4	4	Male	+	+	-	+	+
V 5	5	Male	+	+	-	+	-
N 1	10	Female	+	+	-	-	+
N 2	6	Male	+	+	-	+	+
N 3	5	Female	+	-	+	-	-
N 4	6	Male	-	-	+	-	-
N 5	10	Female	-	-	+	-	-

B: Bacterial meningitis; V: Viral meningitis

**Table 2:** Laboratory test results of all samples

Case no.	CSF				Blood		Culture
	Glucose mmol/L	Protein g/L	Neutrophil 10^6^/L	Leucocyte 10^6^/L	Neutrophil 10^9^/L	Leucocyte 10^9^/L	
B 1	0.02	1.00	NA	3610	7.64	8.96	*H. influenzae*
B 2	2.50	0.28	3	91	8.26	15.8	*E. coli*
B 3	2.96	1.08	35	399	3.74	8.1	*P. aeruginosa*
B 4	1.7	2.48	17,731	21,864	19.54	24.05	*Salmonella*
B 5	1.4	3.69	3756	4172	2.21	4.4	*S. pneumoniae*
V 1	3.24	0.24	8	169	4.16	7.4	EB
V 2	3.79	0.28	10	103	5.77	12.05	EB
V 3	3.59	0.22	3	59	8.52	13.6	*Coxsackievirus*
V 4	3.35	0.21	6	28	9.75	12.4	*Coxsackievirus*
V 5	3.61	0.23	6	35	6.59	8.5	*Coxsackievirus*
N 1	2.61	0.51	15	62	3.1	5.3	-
N 2	4.1	0.27	0	2	3.7	4.9	-
N 3	NA	NA	NA	NA	8.41	11.4	-
N 4	NA	NA	NA	NA	6.09	7.0	-
N 5	3.88	0.19	0	3	7.31	10.8	-

CSF: Cerebrospinal fluid; *H. influenzae*: *Haemophilus influenzae*; *E. coli*: *Escherichia coli*; *P. aeruginosa*: *Pseudomonas aeruginosa*; *S. pneumoniae*: *Streptococcus pneumoniae*; EB: Epstein-Barr virus

### RNA extraction and library sequencing

According to the manufacturer’s instructions, the total RNA was extracted from blood samples using PAXgene blood RNA kit (BD Biosciences, USA). RNA integrity was evaluated by 1% agarose gel electrophoresis, and RNA concentrations were measured using NanoDrop 2000 spectrophotometer (Thermo Scientific, USA). The Ribo-zero rRNA Removal Kit (Illumina, USA) was utilized to construct the lncRNA library to eliminate rRNA from the total RNA. Then, the RNA, after undergoing the Agilent 2100 (Agilent, USA) quality inspection, was used to generate libraries with the NEB Next Ultra™ Directional RNA Library Prep kit (New England Biolabs, USA) for Illumina. Agilent 2100 and ABI StepOnePlus Real-Time PCR System (Applied Biosystems, USA) evaluated the quality of libraries. Finally, the libraries were sequenced on DNBSEQ-G400 (MGI Tech Co., Ltd, China) for 100 bp paired-end sequencing.

### Bioinformatic analysis

Fastp filtered raw data with a Q score lower than 20 and an N count more significant than 10%. Filtered high-quality reads were submitted for further analysis. Afterward, the clean data was mapped to GRCh38 within the Ensembl database using Hisat2 (V 2.1.0, https://daehwankimlab.github.io/hisat2/). Stringtie (V 1.3.3b, http://ccb.jhu.edu/software/stringtie/) was applied to calculate Fragments Per Kilobase per Million (FPKM) to quantify gene expression. Next, DEseq2 was performed to identify differentially expressed mRNAs and lncRNAs. The criteria used for screening were p-adjust < 0.05 and|log2 foldchange| > 1. The results were visualized using R software (V 4.0.5).

### Functional enrichment analysis

Differentially expressed mRNAs were subjected to Pathway and Gene ontology (GO) analysis to determine the roles of these mRNAs in biological processes, molecular function, and cellular component terms using DAVID database (https://david.ncifcrf.gov/). The filtration of the parameter was p-value < 0.05.

### Construction of lncRNA-mRNA co-expression network

The co-expression network was established according to correlation analysis of expression between lncRNA and protein-coding gene. We adopt 0.8 as the Pearson correlation coefficient and p-values < 0.5 for this study. Cytoscape was used to generate the connected network to create a visual representation [15]. Next, the GO and KEGG functional enrichment analysis was performed for mRNAs co-expressed with differential lncRNAs.

### Construction of the ceRNA (lncRNA-miRNA-mRNA) network

The hypothesis of ceRNA proposed that lncRNAs could interact with miRNA sponges to regulate mRNA activity directly. To explore the potential relationship between lncRNAs and mRNAs, miRWalk (http://mirwalk.umm.uni-heidelberg.de/interactions/) was selected to predict mRNA-miRNA interactions. The interaction of lncRNA-miRNA was speculated by NPInter (http://bigdata.ibp.ac.cn/npinter4). The intersection between mRNA-miRNA pairs and lncRNA-miRNA pairs was obtained to get miRNAs. We downloaded the miRNA database GSE131708 from the GEO database to obtain the expression values of miRNAs. The significantly different expression miRNA with a negative correlation of both mRNA and lncRNA were selected. Then, the ceRNA network was constructed.

## Results

### Meningitis exhibited notable alterations in lncRNA and mRNA

To explore the potential role of lncRNAs and mRNAs in meningitis, peripheral blood was analyzed by RNA sequencing from meningitis patients (five bacterial meningitis patients and five viral meningitis samples) and five healthy controls. An average of 14.5 million raw reads and 12.0 million clean reads were obtained from 15 samples. Afterwards, the clean reads were utilized for further analysis. 122,786 RNAs were generated per sample, with 35,422 lncRNAs and 87,364 mRNAs. Out of the 35,422 lncRNAs, 11,134 were found in the bacterial meningitis samples, 11,368 in the viral meningitis groups, and for 87,364 mRNAs, 29,140 were detected in the bacterial meningitis groups and 29,571 in the viral meningitis subjects.F.

**Fig. 1:**
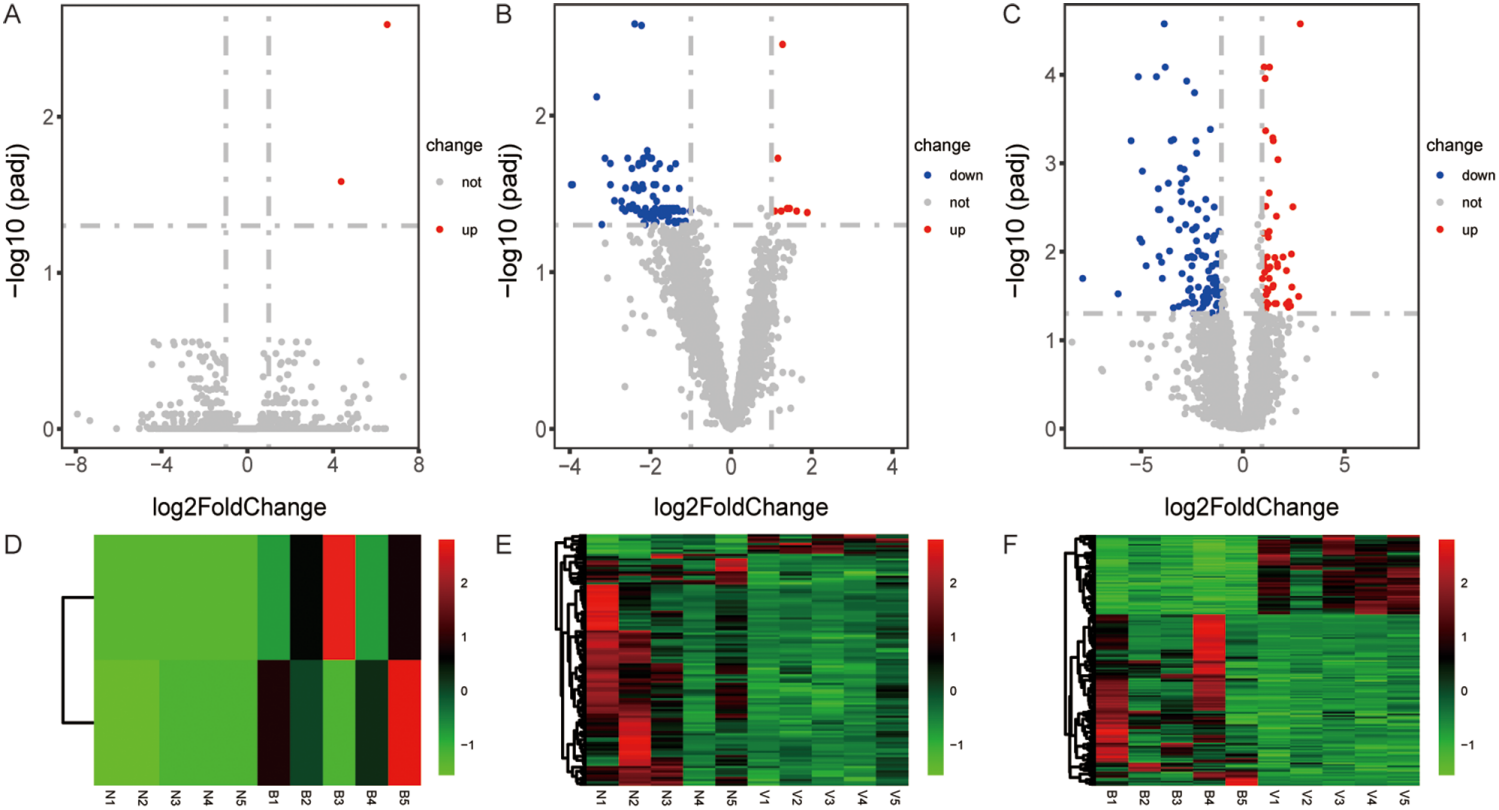
Differentially expressed lncRNA between bacterial meningitis (*n* = 5), viral meningitis (*n* = 5), and standard groups (*n* = 5). Volcano plots were used to visualize lncRNA expression between bacterial meningitis vs. controls **(A)**, viral meningitis vs. healthy samples, and viral meningitis vs. bacterial meningitis **(C)**. Hierarchical clustering **(D, E,** and **F)** showed the lncRNA expression patterns. The red and blue dots were represented up and down represtively lncRNAs. The grey dots showed not significantly expressed lncRNAs

**Fig. 2:**
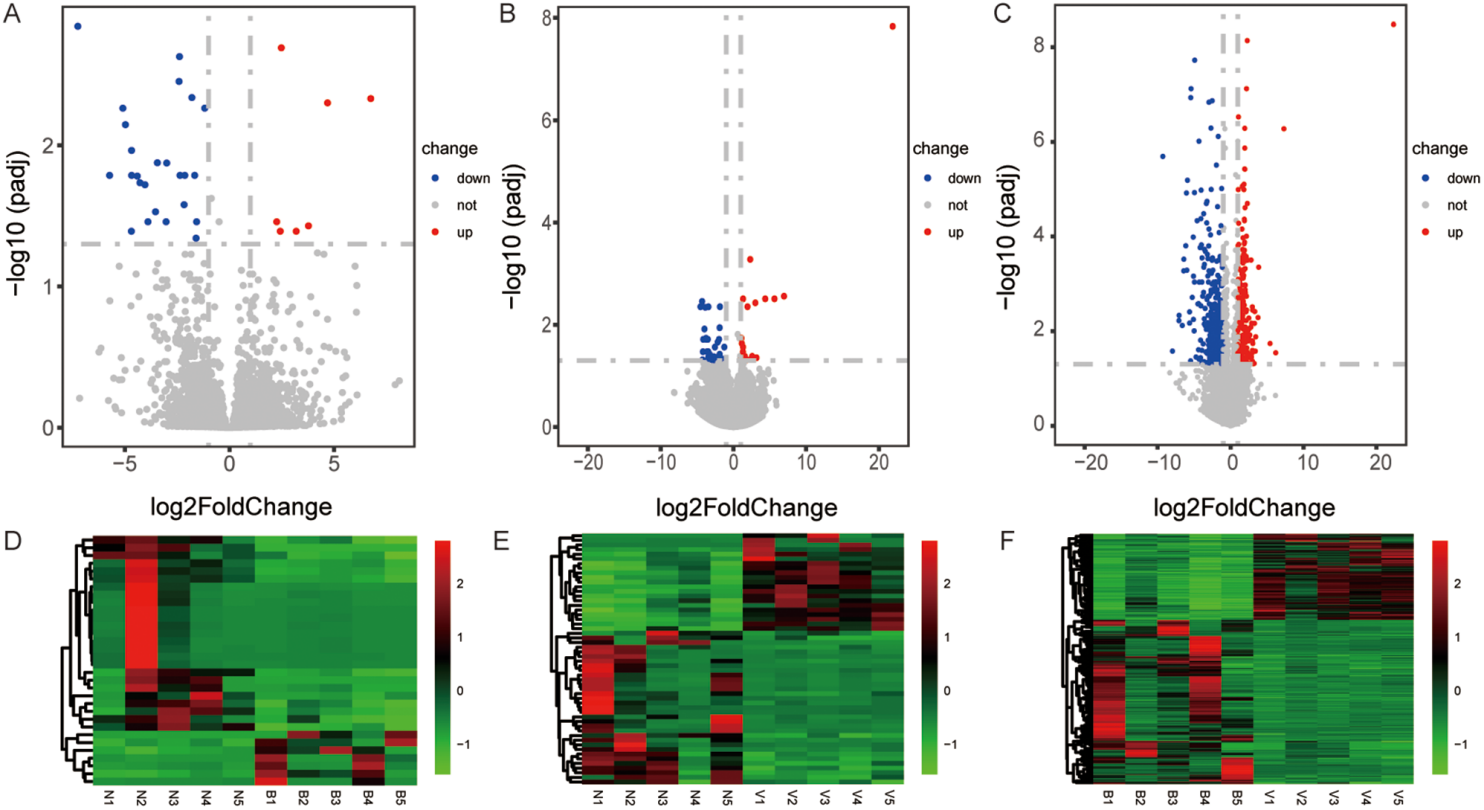
Differentially expressed mRNA between bacterial meningitis (*n* = 5), viral meningitis (*n* = 5), and standard groups (*n* = 5). **A**, **B**, and **C** volcano plots showed the differentially expressed mRNA in bacterial meningitis vs. healthy samples, viral meningitis vs. controls, and viral meningitis vs. bacterial meningitis. The red plots showed significantly upregulated mRNAs, and the blue dots represent significantly downregulated mRNAs. The grey dots showed not significantly expressed mRNAs. **D**, **E**, and **F** heat maps represented the hierarchical clustering corresponding to mRNA

To identify the potential molecules associated with meningitis, we compared the expression profiles of lncRNA and mRNA between patients with viral, bacterial, and healthy controls (Figs. 1 and 2). We applied strict criteria in filtering the differentially expressed lncRNAs and genes. Specifically, we only considered those with a p-adjust value less than 0.5 and an absolute value of log2 fold change greater than 1. Compared with control samples, we detected 2 lncRNAs, upregulated, and 32 mRNAs, including up- and downregulated 7 and 25, respectively, in bacterial meningitis patients (Figs. 1A and 2A). In viral meningitis samples, 115 lncRNAs and 54 mRNAs were identified. Among them, 9 lncRNAs and 21 mRNAs were upregulated. In comparison, 106 lncRNAs and 33 mRNAs were downregulated (Figs. 1B and 2B). One hundred sixty-five lncRNAs (53 upregulated and 112 downregulated) and 765 mRNAs (264 upregulated and 501 downregulated) were found in viral meningitis vs. bacterial meningitis groups (Figs. 1C and 2C). Besides, we conducted hierarchical clustering of the lncRNAs and mRNAs expressed differently in bacterial meningitis vs. controls (Figs. 1D and 2D), viral meningitis vs. controls (Figs. 1E and 2E), and viral meningitis vs. bacterial meningitis (Figs. 1F and 2F). The heatmaps indicated differences in lncRNA and mRNA expression patterns between bacterial meningitis and viral meningitis samples.

### Identified specific lncRNAs and mRNAs in bacterial and viral meningitis

**Fig. 3:**
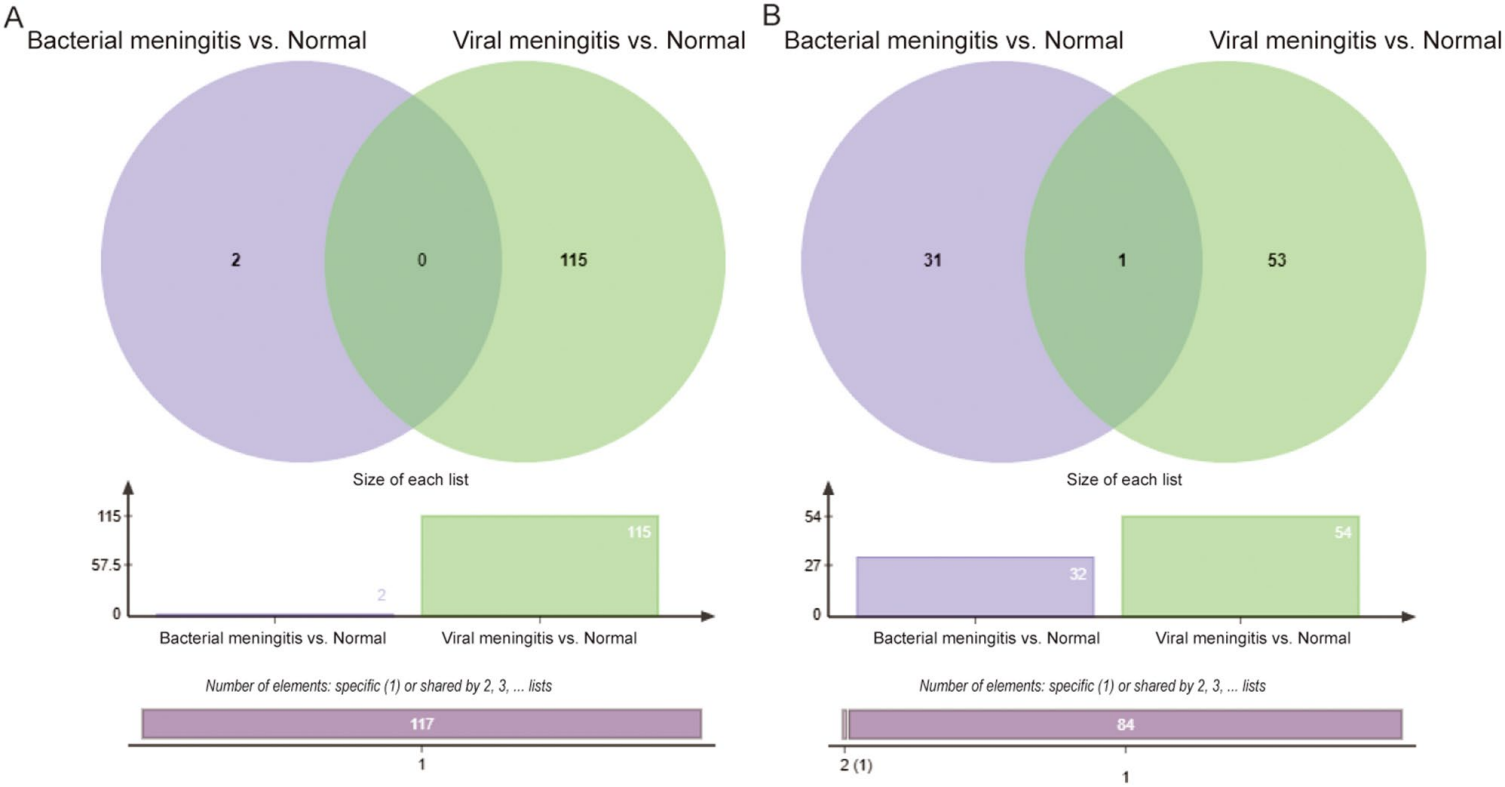
The number of specific lncRNAs and mRNA in bacterial and viral meningitis. **A** showed the signature lncRNAs respectively in bacterial and viral meningitis. The special mRNAs in two meningitis were revealed in **B**

In addition, we identified the specific lncRNAs and mRNAs in bacterial and viral meningitis (Fig. 3). The lncRNAs or mRNAs, which existed in bacterial meningitis vs. controls but not in viral vs. controls, were defined as specific lncRNAs or mRNAs in bacterial meningitis (Fig. 3A). On the contrary, specific lncRNA and mRNA in viral meningitis were gained (Fig. 3B). Then, 2 lncRNAs and 31 mRNAs were obtained in bacterial meningitis, and 115 lncRNAs and 53 mRNAs were obtained in viral meningitis. For mRNA, bacterial and viral meningitis were shared with one mRNA; however, no lncRNA was shared between bacterial and viral meningitis.

### GO and KEGG analysis

Next, the differentially expressed mRNAs were analyzed to discover potential functional implications. In GO and KEGG analysis, 32, 54 and 765 differentially expressed mRNAs were analyzed in bacterial meningitis vs. controls (Fig. S1), viral meningitis vs. control samples (Fig. S2), and viral meningitis vs. bacterial meningitis patients (Fig. S3). The function analysis identified several significantly enriched pathways in this study, including innate immune response, inflammatory response, immune system process, cellular response to interferon-alpha, NF-kappa B signalling pathway, and complement and coagulation cascades. These were linked to inflammatory reactions and other vital cellular processes.

### Specific lncRNA-mRNA co-expression analysis in bacterial and viral meningitis samples

**Fig. 4:**
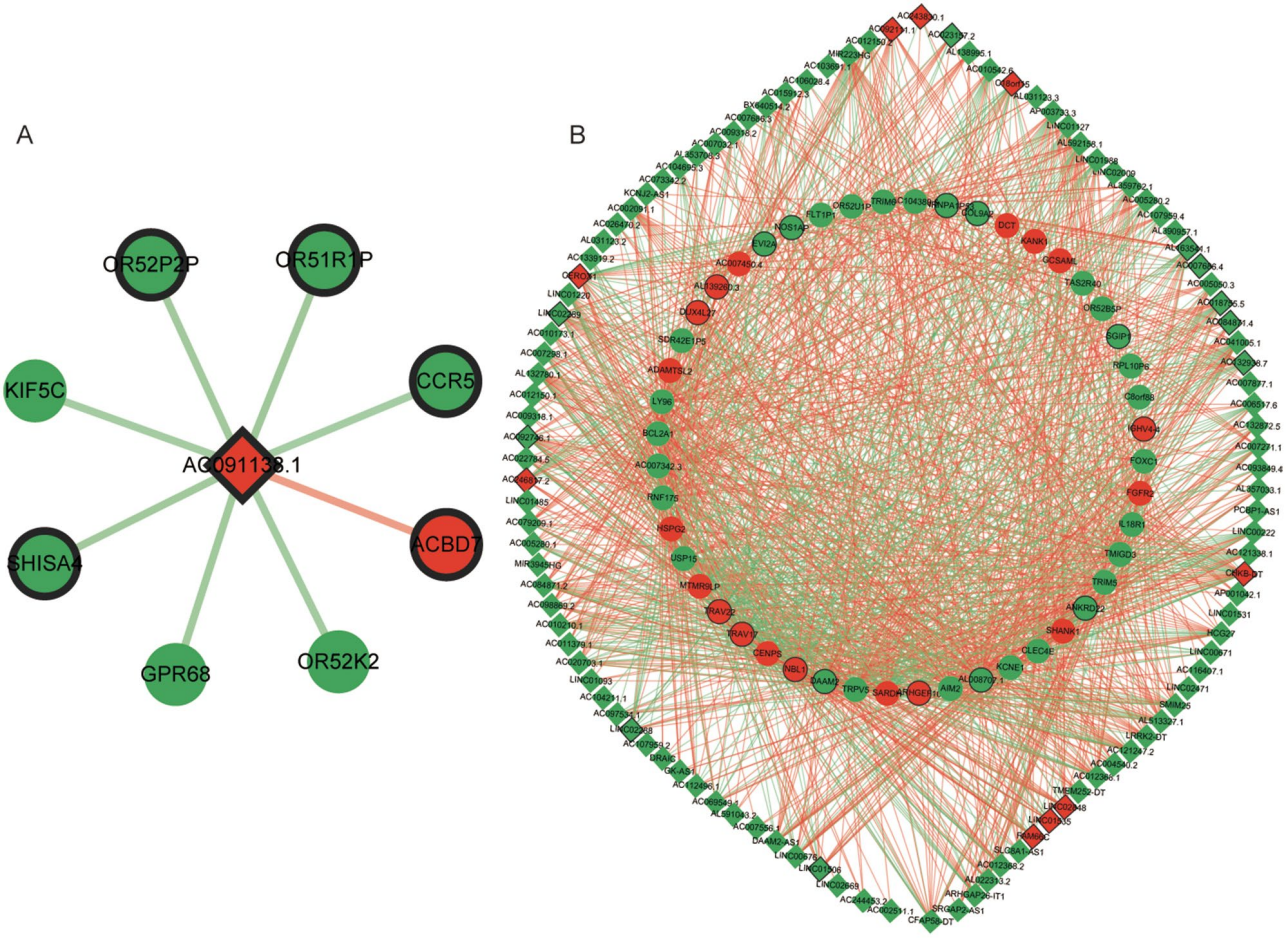
The mRNA-lncRNA co-expression network of mRNA and lncRNA. **A** exhibited the specific co-expression in bacterial meningitis patients. The specific mRNA-lncRNA co-expression network in the viral meningitis group was displayed in **B**. The circle indicated that mRNA and the rhombus represented lncRNA; red and green respectively showed upregulation and downregulation; red lines and green lines expressed positive and negative correlation, respectively; bold outer frame represented the top 10 different expression genes

In bacterial meningitis patients, 8 co-expression relationships were constructed between 8 mRNAs (ACBD7, OR52K2, GPR68, SHISA4, KIF5C, OR52P2P, OR51R1P, and CCR5) and 1 lncRNA (Fig. 4A). In contrast, 1401 co-expression relationships were found between 110 differentially expressed lncRNAs and 49 mRNAs in viral meningitis samples (Fig. 4B). This co-expression network consisted of 892 positive and 509 negative interactions. In addition, our data revealed that a single lncRNA can correlate with 1–30 mRNAs, while one mRNA may correlate with 2-107 lncRNAs. A total of 9 significantly upregulated lncRNAs were identified, including AC243830.1, AC092111.1, CEROX1, AC246817.2, FAM66C, LINC01535, LINC02848, CHKB-DT, and C18orf15.

### CeRNA network analysis in the viral meningitis

**Fig. 5:**
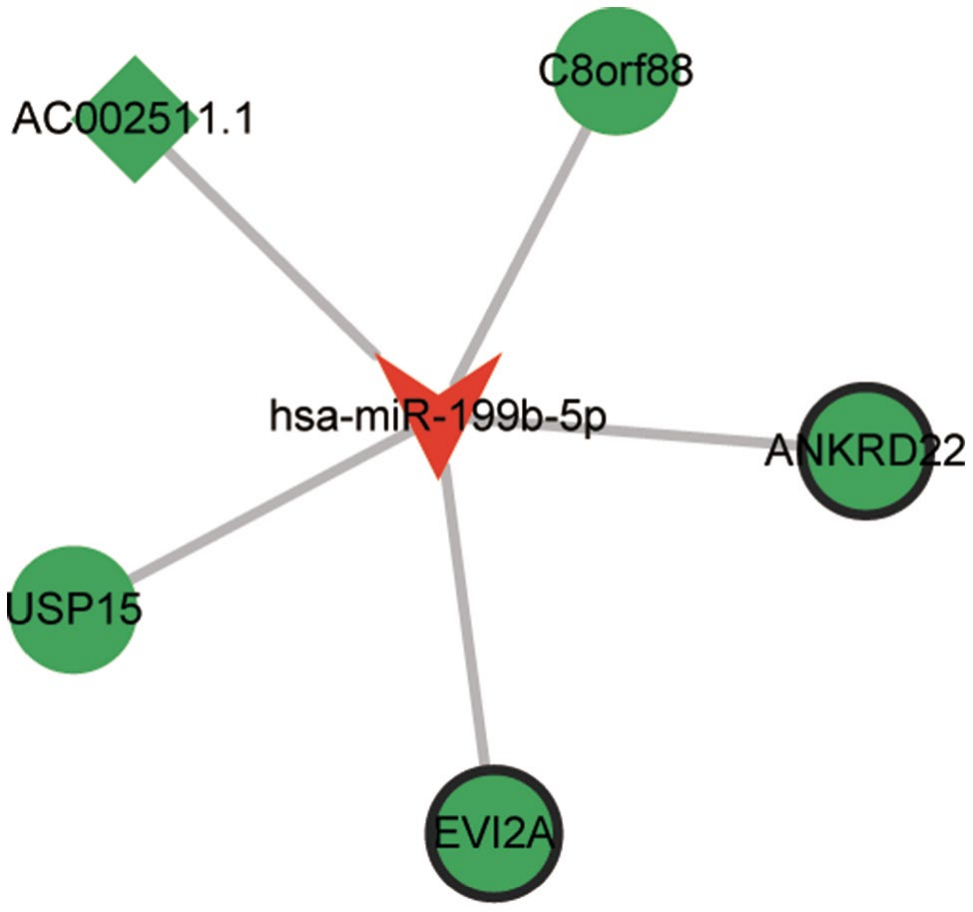
Construction of the regulatory lncRNA-miRNA-mRNA network in viral meningitis. The V type indicated miRNA, the circle represented mRNA, and the rhombus denoted lncRNA. Red and green showed upregulation and downregulation, respectively

Recently, some studies suggested that lncRNAs can function as ceRNAs, competing with mRNAs by binding their common miRNAs in a regulatory circuitry [16–18]. According to ceRNA theory, we constructed the ceRNA networks in bacterial meningitis and viral meningitis samples to investigate whether lncRNA has ceRNA potential in the pathogenesis of bacterial meningitis and viral meningitis. In viral meningitis subjects, the ceRNA network was composed of hsa-miR-199b-5p, 4 mRNAs (ANKRD22, EVI2A, USP15, and C8orf88), and AC002511.1 (Fig. 5). However, the ceRNA networks were not successfully constructed in bacterial meningitis samples.

### ROC analysis identified potential biological markers in bacterial meningitis

**Fig. 6:**
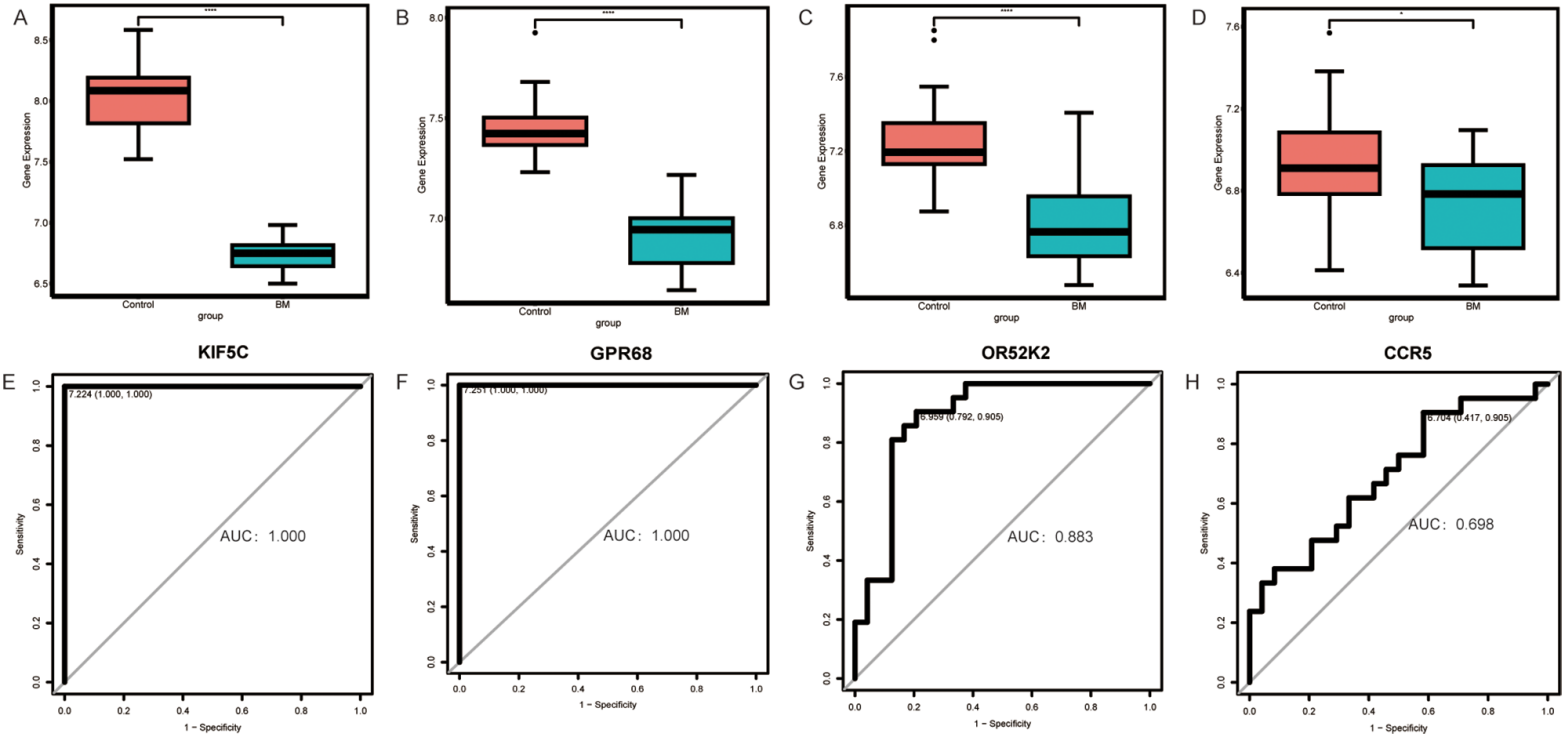
Genes expression and ROC analysis in bacterial meningitis. The box plots indicated the expression of KIF5C **(A)**, GPR68 **(B)**, OR52K2 **(C)**, and CCR5 **(D)**. **E** (KIF5C), **F** (GPR68), **G** (OR52K2), and **H** (CCR5), respectively, represented the results of ROC. AUC, an area under the curve

Eight mRNAs in bacterial meningitis specific lncRNA-mRNA co-expression network were subjected to validate their expression levels in GSE80496, which included 24 bacterial meningitis patients and 21 control samples. The expression of these 8 mRNAs in GSE80496 follows our data. The results are shown in box plots (Fig. 6A-D). Next, those mRNAs were available for the ROC regression analysis. KIF5C and GPR68 had an area under the curve (AUC) of 1.00 (Fig. 6E, F). The AUC of OR52K2 and CCR5 were respectively 0.883 and 0.698 (Fig. 6G, H). The sensitivity and specificity of KIF5C and GPR68 were both 100%. For OR52K2, the sensitivity and specificity were 90.5% and 79.2%, respectively. Meanwhile, the sensitivity and specificity of CCR5 were 90.5% and 41.7%, respectively. For viral meningitis gene verification, we did not find a dataset for viral meningitis in a public database.

## Discussion

A growing body of evidence shows that lncRNA involves diverse biological functions, such as transcriptional activity and interference, epigenetic modification, and other critical regulatory processes [11]. Salisbury et al. suggested that lncRNA Mexis promotes inflammation and contributes to the development of atherosclerosis [19]. LncRNA Mexis promotes the transcription of *Abca1* in macrophages, which gene participates in the production of high-density lipoprotein in atherosclerosis and promotes cholesterol efflux [20]. Moreover, Xu et al. reported that lncRNA RSPH9-4 regulated the permeability probably through the miR-17-5p/MMP3 axis in human brain microvascular endothelial cells [21]. However, the potential regulatory role of lncRNA in meningitis in children is not precise. We profiled the expression of lncRNA and mRNA in bacterial, viral meningitis and healthy controls by RNA-seq analysis. Compared with controls, 32 mRNAs and 2 lncRNAs, and 115 lncRNAs and 765 mRNAs were differentially expressed respectively in bacterial and viral meningitis. Among them, 2 lncRNAs and 31 mRNAs, and 115 lncRNAs and 53 mRNAs were respectively specific in bacterial and viral meningitis. Our results indicated that the atlas of lncRNA and mRNA were distinct among bacterial and viral meningitis and control groups.

The diagnostic value of GPR68, KIF5C OR52K2, and CCR5 were assessed in bacterial meningitis. GPR68 is a member of a novel family of proton-sensing G-protein–coupled receptors [22]. The activity and expression of GPR68 was significantly increased in inflammatory bowel disease [23]. Karki et al. reported that GPR68 suppression was improved in acidosis-induced inflammation and defended bacterial pathogens invasion in lung injuries [24]. C-C Motif Chemokine Receptor 5 (CCR5) belongs to the G-protein-coupled family. It is a 7 transmembrane protein expressed in various cells, e.g., microglia, astrocytes, monocytes, and neurons. In CCR5-silent mice, infection experiments showed that CCR5 is a crucial regulator of neuroinflammatory responses [25, 26]. Le et al. reported that maraviroc, a CCR5 antagonist, can somewhat relieve neuroinflammation [27]. Previously, researchers reported that Olfactory receptors were expressed in macrophages, which participated in inflammatory responses [28]. OR52P2P, OR52K2, and OR51R1P belong to the Olfactory receptors superfamily. Kinesin Family Member 5 C (KIF5C) mutation resulted in neurodevelopmental disorders, including epilepsy, language barrier, and brain malformations [29]. However, KIF5C has not been reported in inflammatory. In this study, CCR5, KIF5C, OR52P2P, OR52K2, and OR51R1P were co-expression with lncRNA AC091138.1 in bacterial meningitis specific co-expression networks. We speculate that AC091138.1 may negatively regulate the above genes to play a certain role in bacterial meningitis. Moreover, KIF5C, GPR68, and OR52K2, with higher AUC, may be potential diagnosis makers in bacterial meningitis.

In viral meningitis specific co-expression networks, a total of 9 significantly upregulated lncRNAs (AC243830.1, AC092111.1, CEROX1, AC246817.2, FAM66C, LINC01535, LINC02848, CHKB-DT, and C18orf15) were identified. Shao et al. found that AC092111.1 might be associated with the prognosis and immune features of patients with glioma [30]. CEROX1 participated in mitochondrial oxidative phosphorylation, strongly associated with inflammation [31, 32]. FAM66C overexpression increased glycolytic activity in human intrahepatic cholangiocarcinoma cell lines [33]. During the inflammatory response, the energy required to activate cells involved in the pro-inflammatory response is primarily achieved through glycolysis and high lactate production [34]. Studies on LINC01535 have focused on cancer progression, such as osteosarcoma, colorectal cancer, and breast cancer [35–37]. The functions of remaining lncRNAs require further exploration. Hence, we speculated that these lncRNAs may play potential roles in viral meningitis. Furthermore, Mukherjee et al. reported that gene regulatory networks had significant alterations with progressive inflammation during autoimmune liver diseases to hepatocellular carcinoma transition [38]. Collecting samples in different inflammation stages to investigate the hub lncRNAs and mRNAs in viral meningitis was essential.

Ankyrin repeat domain-containing protein 22 (ANKRD22), a nucleus-encoded mitochondrial protein, is closely associated with the pathogenesis of multiple diseases, including prostate cancer, gastric mucosal injury, and non-small cell lung cancer and is highly expressed in activated macrophages [39–41]. In gastric mucosal injury, the expression of ANKRD22 was decreased, and the downregulation of ANKRD22 can alleviate the inflammation by activating macrophage and promoting gastric mucosal repair [40]. Another gene, Ubiquitin Specific Peptidase 15 (USP15), which encodes a protease targeting ubiquitin, is critical in regulating innate immune and inflammatory function in response to infectious and tissue damage [42]. It has been documented that viral infection triggers increased interferon signalling when USP15 is lost [43]. The loss of Usp15 function reduces neuroinflammation throughout the body in autoimmune encephalomyelitis (EAE) mice [44]. Sijde et al. found a significant correlation between miR-199b-5p and absolute neutrophil count in removed pancreatic cancer patients [45]. In a cell line related to neuroinflammation, curcumin can reduce neuroinflammation by regulating the miR-199b-5p/IKKb/NF-kB axis in microglia [46]. In this study, the expression of ANKRD22 and USP15 were downregulated in viral meningitis groups, and ANKRD22 expression is similar to the one found in gastritis. CeRNA networks showed that ANKRD22 and USP15 were targets of hsa-miR-199b-5p and lncRNA AC002511.1 were co-expression with hisa-miR-199b-5p in viral meningitis patients. We speculated that the lncRNA AC002511.1 may act as a ceRNA to capture hsa-miR-199b-5p to regulate the expression of ANKRD22 and USP15 in the viral meningitis group.

There are some limitations in our research. Firstly, due to the low morbidity of meningitis, the sample scale is relatively small. Secondly, the pathogens causing meningitis are diverse. We divided the sample into bacterial and viral meningitis. We ignore the heterogeneity of infecting agents. Additionally, construction of the diagnostic model will contribute to distinguishing between bacterial and viral meningitis based on their specific transcriptome features. Finally, further experimental verification in vitro and in vivo is required for lncRNAs involved in meningitis regulation.

## Conclusion

To our knowledge, this is the first attempt to explore the differential lncRNA in children’s peripheral blood in response to bacterial and viral meningitis by RNA-seq. Previous studies have focused on in vitro models. We system compared lncRNA and mRNA expression profiles in bacterial, viral meningitis patients, and healthy controls. We found that lncRNA and mRNA expression profiles significantly changed in patients with meningitis. Moreover, to further explore the cellular heterogeneity within transcriptome differences between viral and bacterial meningitis, conducting single-cell RNA sequencing is necessary for subsequent studies. Our research may provide new insight into understanding the underlying molecular mechanism in meningitis.

### Electronic supplementary material

Below is the link to the electronic supplementary material.

Supplementary Material 1

## Data Availability

Sequence data of this study have been deposited in the Gene Expression Omnibus with accession number GSE248261.
