# Supplementary material for: RNA sequencing reveals differential long noncoding RNA expression profiles in bacterial and viral meningitis in children
Source: BMC Med Genomics. 2024 Feb 12;17:50. doi: 10.1186/s12920-024-01820-y (PMC10863080; doi:10.1186/s12920-024-01820-y)
Supplement: Supplementary file 1 — Supplementary Material 1 [file 12920_2024_1820_MOESM1_ESM.docx]

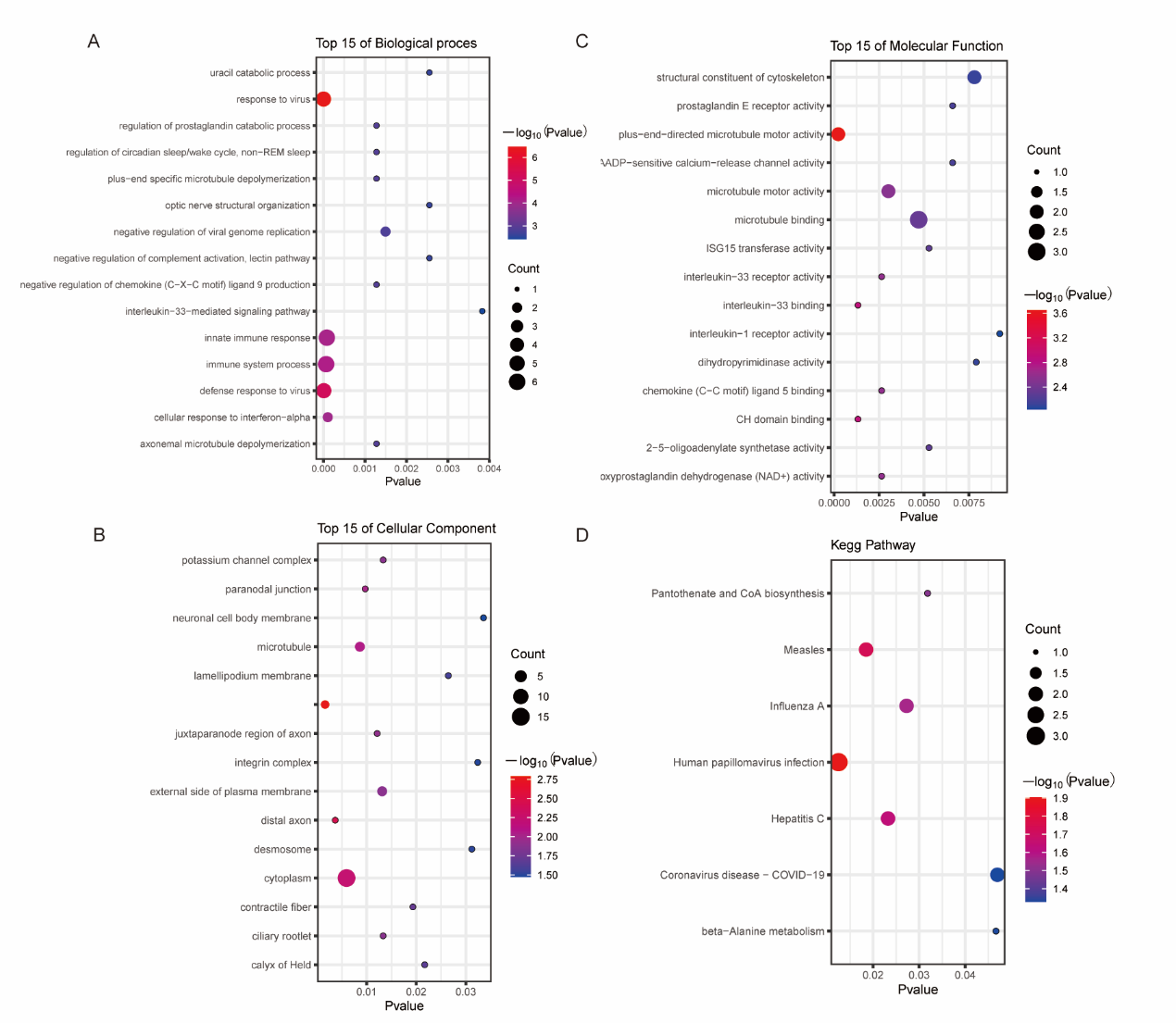


**Figure S1**

The result of mRNA GO (gene ontology) and KEGG (Kyoto Encyclopedia of Genes and Genomes) analysis in bacterial meningitis vs. control groups. A, B, and C represented the top 15 significantly enriched biological processes, cellular components, and molecular functions. D showed that the enrichment results of KEGG.


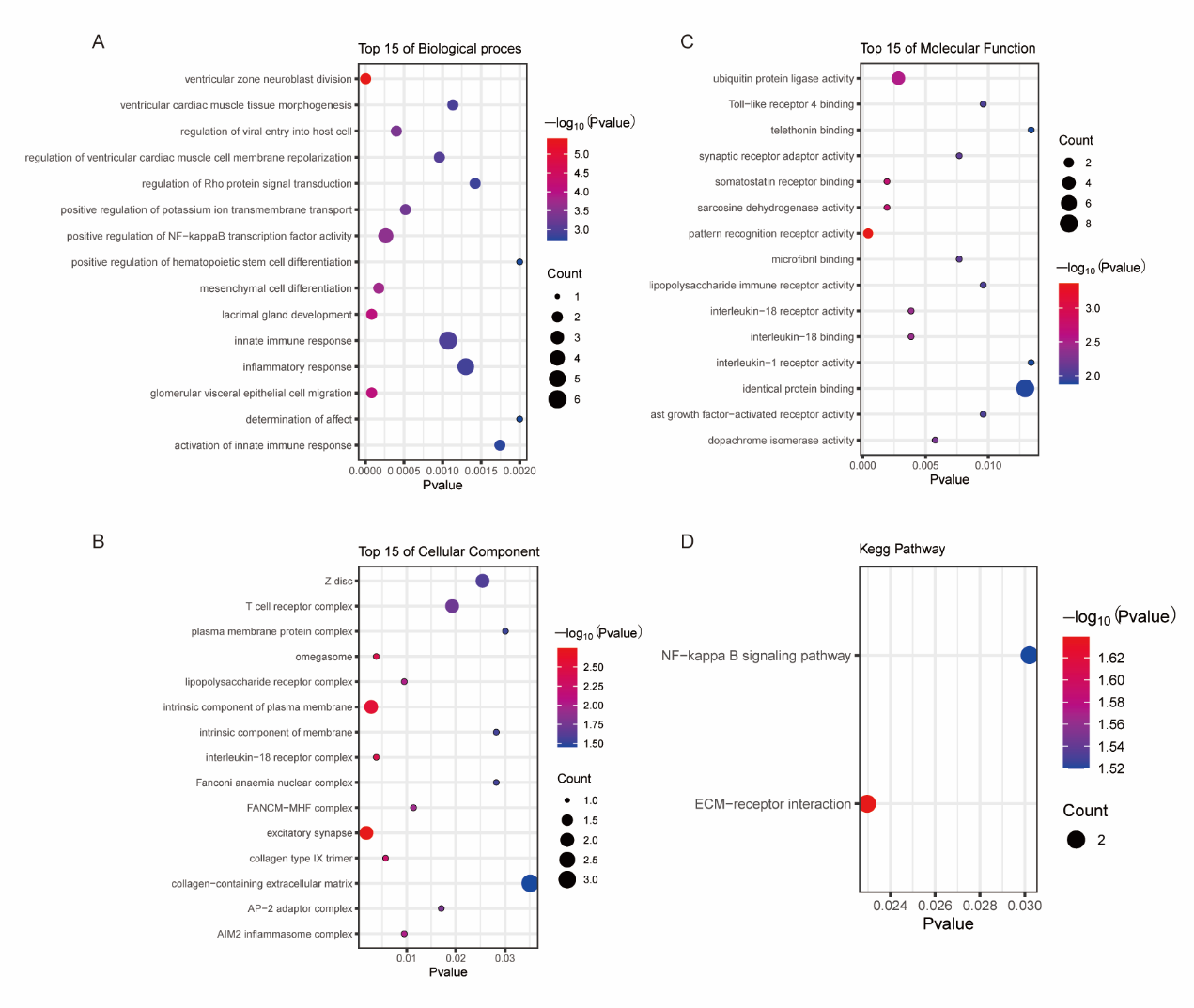


**Figure S2**

GO enrichment result of mRNA in viral meningitis vs. control groups. The top 15 enriched results were displayed in A (biological processes), B (cellular components), and C (molecular functions). The enrichment results of KEGG are shown in D.


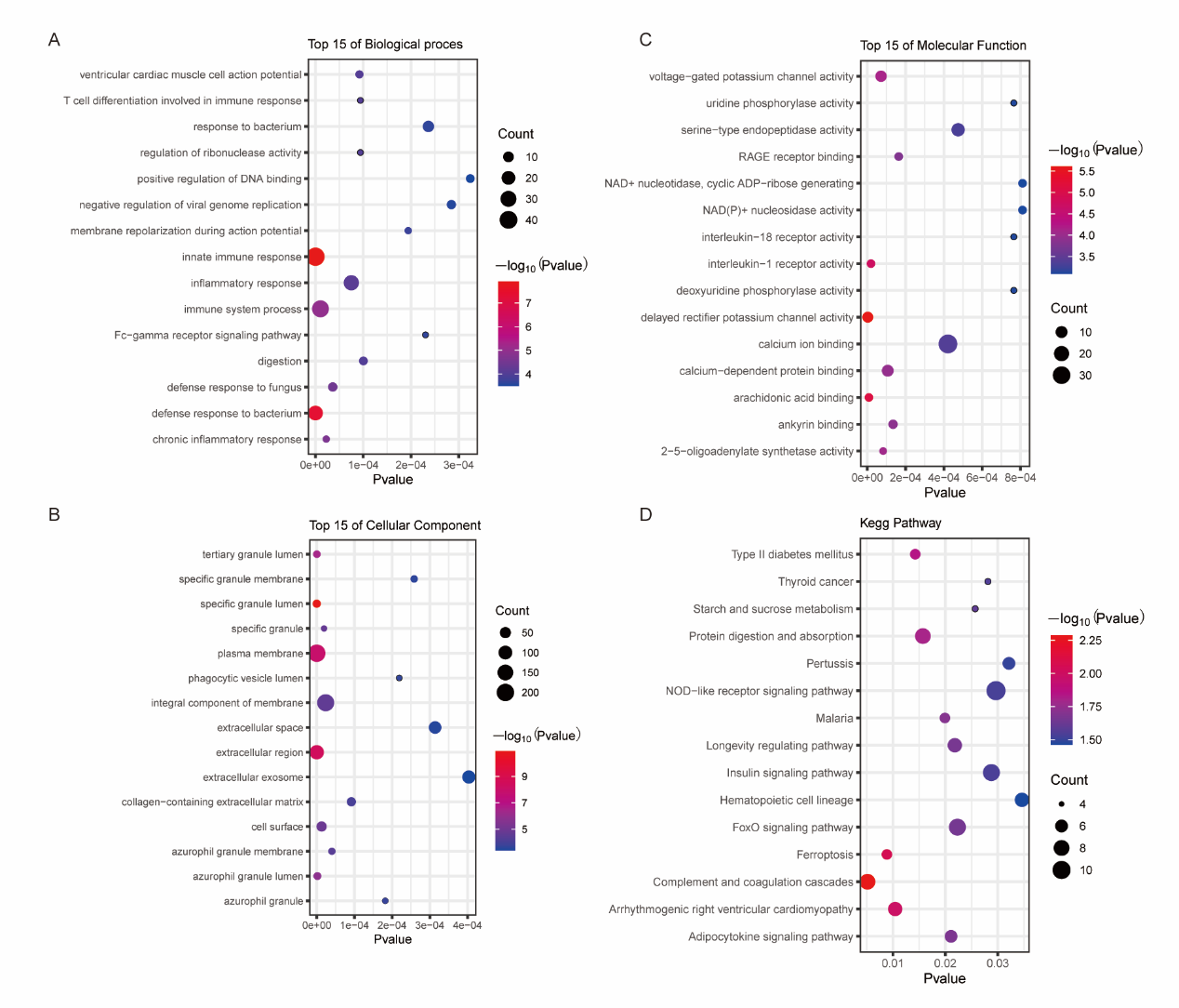


**Figure S3**

The result of mRNA GO and KEGG analysis in viral meningitis vs. bacterial meningitis patients. A, B, and C represented the top 15 significantly enriched biological processes, cellular components, and molecular functions. The enrichment results of KEGG were displayed by bubble charts in D.
